# Transcription associated cyclin-dependent kinases as therapeutic targets for prostate cancer

**DOI:** 10.1038/s41388-022-02347-1

**Published:** 2022-05-14

**Authors:** Theodora A. Constantin, Kyle K. Greenland, Anabel Varela-Carver, Charlotte L. Bevan

**Affiliations:** Imperial Centre for Translational and Experimental Medicine, Department of Surgery and Cancer, Imperial College London, Hammersmith Hospital, London, UK

**Keywords:** Prostate cancer, Transcription

## Abstract

Transcriptional deregulation has emerged as a hallmark of several cancer types. In metastatic castration-resistant prostate cancer, a stage in which systemic androgen deprivation therapies fail to show clinical benefit, transcriptional addiction to the androgen receptor is maintained in most patients. This has led to increased efforts to find novel therapies that prevent oncogenic transactivation of the androgen receptor. In this context, a group of druggable protein kinases, known as transcription associated cyclin-dependent kinases (tCDKs), show great potential as therapeutic targets. Despite initial reservations about targeting tCDKs due to their ubiquitous and prerequisite nature, preclinical studies showed that selectively inhibiting such kinases could provide sufficient therapeutic window to exert antitumour effects in the absence of systemic toxicity. As a result, several highly specific inhibitors are currently being trialled in solid tumours, including prostate cancer. This article summarises the roles of tCDKs in regulating gene transcription and highlights rationales for their targeting in prostate cancer. It provides an overview of the most recent developments in this therapeutic area, including the most recent clinical advances, and discusses the utility of tCDK inhibitors in combination with established cancer agents.

## Introduction

Cyclin-dependent kinases (CDKs) are a family of serine/threonine kinases that are highly conserved across the eukaryotic genome and play fundamental roles in cell cycle regulation and transcriptional control. CDKs are traditionally separated into two major classes: cell cycle-associated CDKs (including CDK1, CDK2, CDK4 and CDK6) and transcription associated CDKs (CDK7, CDK8, CDK9, CDK12, CDK13 and CDK19). Uniquely amongst CDKs, CDK7 is a component of the human CDK-activating kinase (CAK), an important regulator of CDK activity, and has essential roles in both cell division and transcription [1].

Collectively, transcriptional CDKs (tCDKs) orchestrate the transcription cycle, a series of sequential biochemical reactions that control RNA synthesis and processing [2]. Transcriptional dysregulation is increasingly recognised as a hallmark of cancer, with transcription factors (TFs) often acting as oncogenes driving proliferation and survival [3]. This addiction to certain transcriptional programs uncovered new therapeutic vulnerabilities which spotlighted tCDKs as attractive new targets for several types of cancer, including advanced castration-resistant prostate cancer (CRPC). In CRPC, the transcriptional addiction to the androgen receptor (AR, a ligand-activated transcription factor and main therapeutic target) seen in early-stage disease remains a major driver of tumour growth, but available androgen deprivation therapies, all of which effectively target the ligand binding domain of AR, fail to prevent disease progression [4]. Relapse and metastasis are the major cause of death in patients, highlighting an unmet need for novel treatment strategies in CRPC. In this context, targeting of tCDKs, which play crucial roles in gene transcription as well as AR signalling, provides a novel therapeutic strategy for both hormone-naïve and castration-resistant disease.

## Role of tCDKs in eukaryotic transcription

**Fig. 1:**
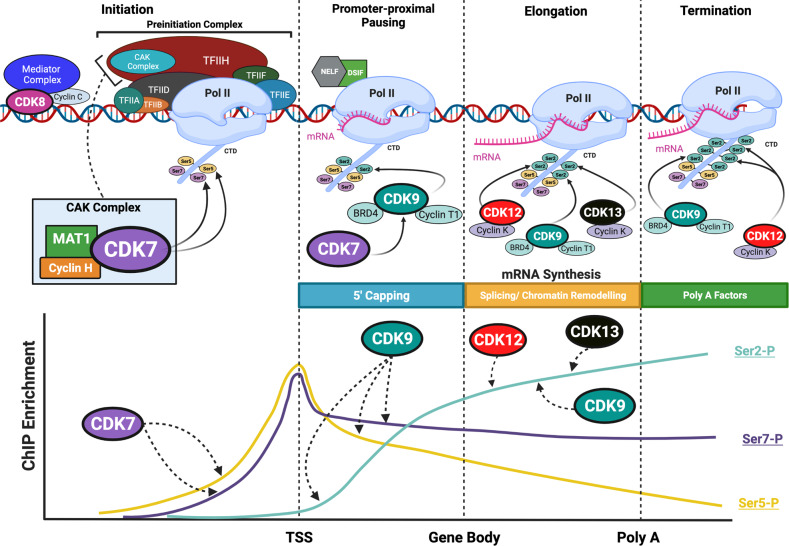
Transcription associated CDKs and their roles in regulating RNA polymerase II (Pol II) during eukaryotic transcription. The Pol II transcription cycle can be divided into four main sequential steps. Initiation begins with the assembly of the preinitiation complex, including Pol II and basal transcription factors (TF), and binding of CDK8/Mediator complex, which anchors the preinitiation complex to gene-specific upstream enhancers. Promoter escape is facilitated by the phosphorylation of the Pol II C-terminal domain (CTD) at serine 5 (Ser5) and serine 7 (Ser7) by CDK7, which promotes recruitment of pre-mRNA 5′capping enzymes. Transcription is provisionally paused downstream of the transcription start site by the association of two negative factors, DRB sensitivity-inducing factor (DSIF) and negative elongation factor (NELF). Bromodomain-containing protein 4 (BRD4) and the positive transcription elongation factor (P-TEFb), consisting of CDK9 and cyclin T, are recruited to acetylated chromatin. CDK7 activates CDK9 through T-loop phosphorylation, which in turn mediates promoter-proximal pause release by phosphorylating DSIF, NELF, and the Pol II CTD at serine 2 (Ser2). Progression from the transcriptional start site (TSS) across the gene body and productive elongation are maintained by the differential phosphorylation of the Pol II CTD at Ser2, Ser5, and Ser7 by CDK9 and CDK12/CDK13. The pattern of CTD phosphorylation contributes to the recruitment of splicing and chromatin remodelling factors. Termination is regulated by CDK12 which promotes the use of distal 3′ transcription termination sites and recruitment of cleavage and polyadenylation (Poly A) factors. The graph underneath depicts relative abundance of Pol II Ser-CTD modifications across protein-coding genes, determined by chromatin immunoprecipitation (ChIP)-seq investigations. Created with BioRender.com.

Transcription of protein-coding genes is mediated by RNA polymerase II (Pol II). Pol II is a multiprotein complex that transcribes DNA into mRNA via a series of highly coordinated events, collectively known as the transcription cycle. Broadly, the Pol II transcription cycle can be divided in four stages: assembly of the preinitiation complex, promoter escape, elongation, and termination [2]. The coordinated activity of tCDKs allows an orderly transition between the stages of the transcription cycle; they regulate transcription by phosphorylating the carboxy-terminal domain (CTD) of RPB1, the largest subunit of Pol II, as well as other TFs (Table 1). The Pol II CTD is composed of up to 52 heptapeptide repeats (Tyr-Ser-Pro-Thr-Ser-Pro-Ser) and serves as a binding structure for the other nuclear factors involved in transcription [5, 6]. Patterns of CTD phosphorylation differ at different stages of transcription and allow for the timely recruitment of factors important for mRNA elongation and maturation (Fig. 1) [6].

**Table 1:** Substrates of transcription associated CDKs.

Transcriptional CDK (partner cyclin/other partner protein)	Substrate(s)	Residue(s)	Downstream effect(s)	Reference(s)
CDK7 (cyclin H, MAT1)	RPB1 (C-terminal domain)	Ser5	Facilitates promoter escape	[7, 8]
	RPB1 (C-terminal domain)	Ser7	Recruitment of RPAP2, transcription of snRNA genes	[9]
	CDK9 (T-loop domain)	Ser175	Promotes recruitment of BRD4	[10]
		Thr186	Enhanced activity	[11]
	CDK12 (T-loop domain)	Thr893	Enhanced activity	[12]
	CDK13 (T-loop domain)	Thr871	Enhanced activity	[12]
	MED1 (C-terminal region)	Thr1457	Recruitment to chromatin, association with AR and the transcription machinery	[13]
	SF3B1	18 putative sites (residues 207-434)	Affects association with splicing speckles	[12]
	U2AF2	Unknown	Unknown	[12]
	DNA-binding TFs: AR, E2F1, ERα, Ets1, p53, PPARα, PPARγ2, RARα, RARγ, YAP/TAZ, SF1	Ser515 AR, Ser403/Thr433 E2F1, Ser118 ERα, Thr38 Ets1, Ser33 p53, Ser112 PPARα, Ser12/Ser21 PPARγ2, Ser77 RARα, Ser77/Ser79 RARγ, Ser128/90 YAP/TAZ, Ser203 SF1	Promotes activity and/or regulation of protein turnover	[14]
CDK8/CDK19 (cyclin C)	Several validated DNA-binding TFs, Mediator subunits, and chromatin regulators (e.g. STAT1, MED12, MED13, SIRT1)	Ser727 STAT1, Ser688 MED12, Ser749 MED13, Thr530 SIRT1	Context-dependent effects; potentiation of transcriptional activation	[15]
CDK9 (cyclin T1)	RPB1 (C-terminal domain)	Ser2	Promotes transcription elongation	[16]
	DSIF (Spt5 subunit)	Thr4	Facilitates promoter-proximal pause release of Pol II	[17]
	NELF (NELF-E subunit)		Facilitates promoter-proximal pause release	[18]
	XRN2	Thr439	Enhanced cleavage of the RNA transcript from Pol II	[19]
	DNA binding TFs: AR, ERα	Ser81, Ser294	Transcriptional activation in response to ligand	[20, 21]
CDK11 (cyclin L)	AR (N-terminal domain)	Ser308	Repression of AR transcription	[22]
CDK12/CDK13 (cyclin K)	RPB1 (C-terminal domain)	Ser2	Promotes elongation and the use of distal 3′ transcription termination sites	[23, 40]
	RPB1 (C-terminal domain)	Ser5		

*CDK* cyclin-dependent kinase, *RPB1* DNA-directed RNA polymerase II subunit rpb1, *MED1* mediator complex subunit 1, *SF3B1* splicing factor 3b subunit 1, *U2AF2*, small nuclear RNA auxiliary factor 2, *TF* transcription factor, *AR* androgen receptor, *ERα* oestrogen receptor alpha; *Ets1* ETS proto-oncogene 1, *PPAR* peroxisome proliferator-activated receptor alpha, *RAR* retinoic acid receptor, *YAP* yes-associated protein 1, *TAZ* transcriptional coactivator with PDZ-binding motif, *SF1* splicing factor 1 *DSIF* DRB sensitivity inducing factor, *NELF* negative elongation factor, *XRN2* 5′-3′ exoribonuclease 2, *RPAP2* RNA polymerase II-associated protein 2, *BRD4* bromodomain-containing protein 4.

### CDK7 is a “master regulator” of transcription

CDK7 regulates Pol II-mediated transcriptional initiation and pausing, in addition to indirectly promoting transcript elongation via other tCDKs through its CAK activity. In eukaryotes, transcription initiation begins with the assembly of the preinitiation complex. The minimal preinitiation complex includes Pol II and six general TFs: TFIIA, TFIIB, TFIID, TFIIE, TFIIF, and TFIIH [7]. TFIIH consists of two subcomplexes: the core complex, comprising ATP-dependent helicases, and the CAK complex, which harbours the kinase activity of CDK7 [8]. In addition, the Mediator complex, which is generally required for transcription by Pol II, anchors the preinitiation complex to gene-specific upstream enhancers [9]. At the transcription start site (TSS), following DNA unwinding by helicases, Pol II must be released from the preinitiation complex and dissociate from the Mediator complex. This process, termed promoter escape, is facilitated by the CDK7-dependent phosphorylation of Ser5 in the Pol II CTD [10, 11] (Table 1). CDK7 also phosphorylates Ser7-Pol II CTD. The exact function of this remains unclear, but evidence suggests it facilitates recruitment of RPAP2, a Pol II-associated Ser5 phosphatase, which in turn recruits the Integrator complex required to regulate transcription of small nuclear RNA (snRNA) genes [12]. Upon promoter escape, phosphorylation of Ser5-Pol II CTD by CDK7 promotes the recruitment of capping enzymes which mediate 5’ capping of the nascent pre-mRNA. Consistently, inhibition of CDK7 causes diminished recruitment of capping enzymes and reduction in capped transcripts [13].

The roles of CDK7 extend past the transcription initiation stage. Following promoter escape, transcription is paused approximately 50-70 nucleotides downstream of the TSS. This event, termed promoter-proximal pausing, serves as a regulatory mechanism perhaps especially for genes important for development and cell cycle progression and also ensures accurate processing of RNA and maturation of the elongation complex [1, 14]. CDK7 indirectly mediates promoter-proximal pause release by phosphorylating the T-loop of CDK9, a component of the positive transcription elongation factor b (P-TEFb) [15, 16]. P-TEFb activity is required for Pol II maturation and transition into the productive elongation stage [17]. The phosphorylated forms of the Pol II CTD mediate interactions with histone methyltransferases, thereby regulating transcription associated epigenetic modifications. Specifically, phosphorylation of Pol II CTD by CDK7 has been shown to activate the SETD1A/B histone H3K4 methyltransferase complex and regulate H3K4me3 spreading into gene bodies [18]. Since trimethylated H3K4 modifications play an important role in pre-mRNA splicing [19], it is possible that CDK7 also regulates several splicing and RNA processing factors. Using quantitative phosphoproteomics, a recent study showed that the largest subunit of the spliceosome factor 3b, complex, SF3B1, and the U2 auxiliary factor, U2AF2, are CDK7 substrates [20]. Further supporting this, inhibition of CDK7 activity using the covalent inhibitor SY-351 induced widespread changes in alternative mRNA splicing in vitro [20].

TFIIH also functions in nucleotide excision repair (NER). TFIIH is recruited to sites of DNA damage, where it promotes incision and exclusion of DNA bases. Then, xeroderma pigmentosum A catalyses the release of CAK from the core TFIIH complex, facilitating effective NER of damaged bases [21]. Following repair, CAK re-associates with TFIIH, enabling transcription to proceed [22]. Previous research has shown that inhibition of CAK activity improves repair efficiency, indicating that CAK may be a negative regulator of NER [23].

### CDK9 stimulates transcript elongation

As alluded to above, CDK9 associates with cyclin T1 to form P-TEFb, a potent general transcription factor that is maintained under stringent negative regulation by the 7SK small nuclear ribonucleoprotein (snRNP) [24]. The canonical 7SK snRNP complex contains the highly abundant non-coding 7SK snRNA, which is stabilised by La Ribonucleoprotein 7 (LARP7) and methylphosphate capping enzyme (MePCE). The 7SK snRNP complex functions as a scaffold where P-TEFb is inactivated through association with hexamethylene-bis-acetamide inducible proteins 1/2 (HEXIM1 and/or HEXIM2) [25]. The chromatin-tethered 7SK snRNP complex represents a major reservoir of transcriptionally inactive P-TEFb and serves as a source of P-TEFb to facilitate Pol II escape. Release of P-TEFb occurs when Bromodomain-containing protein 4 (BRD4) is recruited to TSS via histone acetylation and competes with the inhibitory HEXIM/7SK complex [26].

Promoter-proximal pausing of Pol II is initiated by the association of two negative factors, DRB sensitivity-inducing factor (DSIF) and negative elongation factor (NELF) [27]. P-TEFb phosphorylates the Spt5 subunit of DSIF, converting it to a positive elongation factor [28] and causing dissociation of NELF from Pol II [27]. The transition to productive elongation is then mediated by P-TEFb, which phosphorylates Ser2 residue on the CTD of Pol II [29] (Table 1). Additionally, CDK9 also phosphorylates Thr4 of the Pol II CTD; this is required for histone mRNA 3′ end processing by facilitating the recruitment of 3′ processing factors to histone genes [30].

Emerging evidence suggests a role of CDK9 in regulating transcription termination (Table 1). A high-throughput screen for CDK9 substrates identified over 100 proteins, a large majority of which were implicated in transcription and RNA catabolism [31]. Xrn2, a nuclear 5′-to-3′ exoribonuclease required for Pol II termination, was validated as a CDK9 substrate, suggesting that CDK9 directly regulates a transcription termination pathway. CDK9 activity is also essential for the deposition of histone H2B monoubiquitylation, through a CTD-dependent mechanism [32]. In its absence, Pol II continues transcription until an alternative downstream polyadenylation site is reached. Furthermore, CDK9 inhibitors decrease transcription downstream of the polyadenylation site, supporting the idea that CDK9 activity plays a role in transcription termination [33] (Fig. 1).

### CDK8 and CDK19 are Mediator-associated kinases

CDK8 is a transcriptional kinase which associates with regulatory genomic elements together with the Mediator complex. CDK8 forms the CDK8 kinase module together with cyclin C, MED12, and MED13. Cyclin C and MED12 activate the CDK8 kinase function, while MED13 enables association with the Mediator complex [34]. Three components of the CDK8 module, namely CDK8, MED12, and MED13, have paralogs that have arisen from gene duplications. These are CDK8-like (CDK19), MED12-like (MED12L), and MED13-like (MED13L), respectively. These paralogs can form part of the CDK8 kinase module but are mutually exclusive of each other, enabling the assembly 8 different CDK modules, always together with cyclin C [35]. Importantly, the existence of several CDK-Mediator complexes indicates potential functional distinctions.

As the Mediator complex is usually required for transcription by Pol II in mammalian cells [9], disruption of CDK8/CDK19 function would be predicted to have a global effect on transcription. However, genetic and pharmacological inhibition of CDK8/CDK19 has been shown to affect only subsets of genes, which differ between cell types [36, 37]. It could be speculated that this cell type-specificity of Mediator kinases is determined by chromatin structure and histone modifications, which give rise to cell type-specific enhancers, TF binding patterns, and enhancer-promoter communication. Supporting this idea, in a phosphoproteomics study, inhibition of CDK8/CDK19 revealed that many substrates are DNA-binding TFs or chromatin regulators [38] (Table 1).

### CDK12 and CDK13 multi-task to tune transcription

CDK12 and CDK13 are two evolutionarily-related kinases that associate with cyclin K and display Pol II CTD kinase activity, particularly when Ser7 CTD residue is pre-phosphorylated [39]. Despite the structural similarity of their kinase domain and partnering with the same cyclin, CDK12 and CDK13 regulate distinct biological processes. CDK12 inhibition primarily affects the expression of genes involved in the DNA damage response, although suppression of super-enhancer-associated genes has been observed at high inhibitor concentrations [40]. CDK12 also affects the processing of nascent RNA, by promoting the use of distal 3′ transcription termination sites and suppressing intronic polyadenylation, resulting in the production of full-length gene transcripts [41]. Notably, genes implicated in the DNA damage response are longer and display more intronic polyadenylation sites compared to other expressed genes [42], which could explain how depletion of CDK12 activity selectively alters their expression. Emerging evidence also suggests a role for CDK12 in controlling splicing and regulating the expression of splicing factors. CDK12 has been shown to interact with several components of the core spliceosome and regulators of constitutive and alternative splicing and has even been proposed as a bona fide component of the splicing machinery [43]. Taken together, loss of CDK12 activity affects transcription elongation, RNA processing, and termination, and may also lead to splicing defects and genome instability, although the mechanism underlying the specificity for certain genes requires further research.

The role of CDK13 in transcription is less well understood but recent evidence shows that it is distinct from that of CDK12. Although it has been reported to have Pol II CTD kinase activity (Fig. 1), knockdown of CDK13 leads to less CTD phosphorylation alteration than knockdown of CDK12 [39]. Furthermore, research indicates CDK13 preferentially regulates snRNA and small nucleolar RNA genes that guide posttranscriptional modifications of other RNAs, primarily ribosomal RNAs, as well as some genes involved in mitochondrial energy metabolism [44].

### Other transcriptional CDKs

CDK10 is activated by the partner cyclin M and controls transcription mediated by ETS2, a member of the ETS family of oncogenic TF, by phosphorylation and promoting proteasomal degradation [45, 46]. Therefore CDK10/cyclin M can negatively regulate ETS2 transcription, suggesting a tumour suppressor role. CDK11 is activated by L-type cyclins and has roles in alternative splicing, where it can influence splice site selection [47]. Additionally, CDK11 has an important role in regulating the expression of replication-dependent histone genes by promoting elongation and 3′-end processing [48]. Replication-dependent histone genes have fundamental roles in cell division, suggesting CDK11 may have an important role in cancer.

## Rationale for targeting tCDKs in CRPC

Transcription deregulation is a feature of most cancer types and is increasingly regarded as a hallmark of cancer. Dysregulated transcriptional programs are established early during tumorigenesis and continue to operate after a malignant state is reached. Acquisition of aberrant gene regulation, coupled with the gain of hyperactive regulatory elements (e.g., super-enhancers) and associated increase in transcription rates provide transformed cells with a growth and survival advantage. This state in which cancer cells show high dependency on dysregulated transcriptional programs is termed transcriptional addiction. Emerging evidence suggests transcriptionally addicted cancer cells are especially sensitive to transcriptional disruptions, highlighting transcriptional addiction as a potential therapeutic vulnerability [3].

Most prostate cancers display transcriptional addiction to AR, a member of the steroid hormone nuclear receptor family. The AR protein consists of three functional domains which are required for normal receptor function: the N-terminal domain, the DNA binding domain, and the C-terminal ligand binding domain. Upon androgens binding to the ligand binding domain, cytoplasmic AR is released from molecular chaperones and is shuttled to the nucleus. The DNA binding domain tethers AR to chromatin at androgen response elements located in the promoters and/or enhancers of AR target genes. The transcriptional activity of AR is then regulated through interaction with AR coregulators, which recruit chromatin-modifying enzymes that either promote a state of open chromatin, facilitating recruitment and assembly of the Pol II transcriptional machinery (co-activators), or promote chromatin condensation, thereby impeding transcription (co-repressors) [4]. Rewiring of the AR transcriptional program occurs early during prostate epithelial transformation. *ETS* gene rearrangements, which effectively place members of the ETS family (commonly *ERG* and *ETV1*) under the control of AR-regulated promoters (e.g., *TMPRSS2*) are present in ~50% prostate tumours [49]. The resulting androgen-induced overexpression of oncogenic TFs contributes to dysregulated transcription and a poor prognosis [50]. In addition, transcriptional hijacking of AR is accompanied by extensive reprogramming of the AR cistrome, which displays increased AR binding sites, as well as enrichment of the pioneer factor FOXA1 and the homeobox factor HOXB13 at tumour-specific AR binding sites, further promoting oncogenic activation of AR [51].

Systemic inhibition of AR signalling, either by depleting circulating androgens or by impeding ligand-dependent activation through receptor antagonists, remains the gold-standard treatment for metastatic prostate cancer. However, the selective pressure of antiandrogen therapy near-universally leads to development of therapy-resistance termed castration-resistant prostate cancer (CRPC). CRPC cells often acquire point mutations leading to amino acid substitutions in the AR ligand binding domain, amplification of the *AR* gene, and increased expression of intra-tumoral androgen-producing enzymes, facilitating receptor activation even when androgen availability is significantly reduced. Upregulation of AR co-activators and downregulation of AR co-repressors can also contribute to AR signalling reactivation during androgen deprivation therapy. Recently, truncated AR splice variants which lack a ligand binding domain and display constitutive activity even in low/no androgen conditions emerged as a possible mechanism for CRPC tumours to acquire ligand-independent AR signalling [4]. Clinically, the development of castration-resistance limits patient survival to 9-30 months [52], highlighting an unmet need for novel strategies to achieve inhibition of oncogenic transcription and AR signalling in CRPC.

**Fig. 2:**
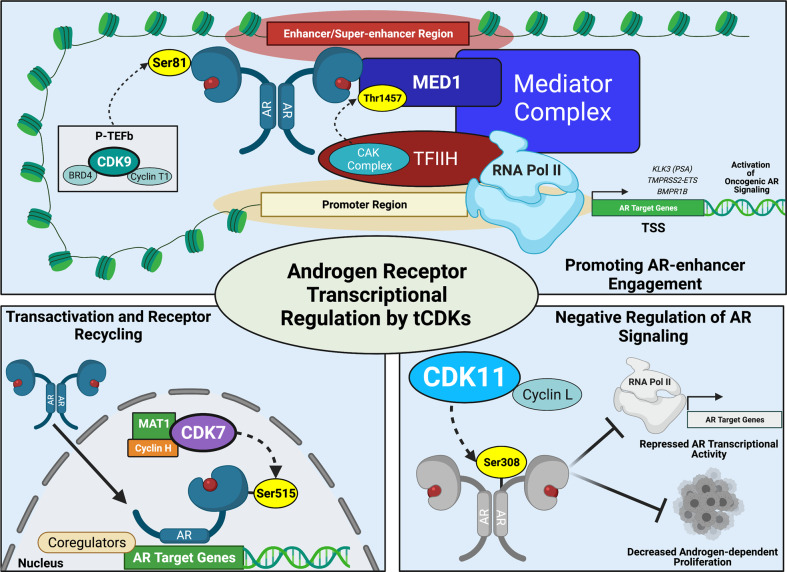
The role of transcription associated CDKs (tCDKs) in regulating the transcriptional activity of the androgen receptor (AR). (Top) CDK7 phosphorylates MED1 at threonine 1457 (Thr1457) and promotes association of the Mediator complex with AR and the transcription machinery. Phosphorylation of chromatin-bound AR at serine 81 (Ser81) by CDK9 enhances recruitment of co-activators and strongly promotes transcription of AR target genes. (Bottom left) CDK7 directly phosphorylates AR at serine 515 (Ser515), which directs efficient recycling of the receptor for cyclical gene activation. (Bottom right) Phosphorylation of AR at serine 308 (Ser308) by CDK11 leads to transcriptional repression through increased recruitment of AR co-repressors. Created with BioRender.com.

The coordinated activity of tCDKs appears to be crucial for the transcriptional activation of AR, indicating that these kinases would be good therapeutic targets for CRPC. Several tCDKs can directly phosphorylate AR to regulate its stability, nuclear localisation, or/and transcriptional output (Table 1 and Fig. 2). Phosphorylation of Ser515 in the N-terminal domain of AR by CDK7 is required for both maximal transactivation and for optimal cyclical ubiquitination, proteasomal degradation and re-recruitment of AR to gene promoters [53, 54]. Recent studies have shown that the Mediator Complex Subunit 1 (MED1, also known as TRAP220/DRP205) can undergo CDK7-dependent phosphorylation at Thr1457 which promotes engagement with AR at enhancer and super-enhancer sites, and transcription of AR regulated genes [55]. Phosphorylation of chromatin-bound AR at Ser81, the major site that is modified in response to androgen treatment, is primarily mediated by CDK9 [56]. Ser81 phosphorylation enhances recruitment of the histone acetyltransferase (and AR coactivator) p300 and of BRD4, which further releases P-TEFb, creating a positive feedback loop that maintains transcription of AR target genes [57]. In addition, CDK11 can phosphorylate AR at Ser308, although this modification has a negative effect on AR-mediated transactivation [58].

Recently published in vivo studies demonstrated the antitumour activity of compounds targeting CDK7, CDK9 and CDK8/CDK19 in CRPC models, with mechanistic explorations consistently highlighting suppression of oncogenic AR transcription as a downstream effect of treatment. Inhibition of CDK7 attenuated AR signaling regardless of enzalutamide sensitivity status, by preventing association with oncogenic super-enhancers in vitro, and led to potent growth suppression in the VCaP CRPC xenograft model, which harbours *TMPRSS2-ERG* gene fusion and expresses several constitutively active AR splice variants [55]. Screening for novel compounds that could modulate the transcriptional output of AR returned a lead, the molecular target of which was determined to be CDK9 [59]. The optimised compound significantly reduced the growth of the 22Rv1 CRPC xenograft model, which displays intrinsic enzalutamide resistance due to a point mutation in the AR ligand binding domain. Similarly, CDK8/CDK19 inhibition downregulated AR activity along with cell cycle processes in vitro, and reduced VCaP xenograft growth in vivo [60]. In addition, CDK12 was identified through a CRISPR screen as a kinase that is essential for prostate cancer cell survival [61]. Suppression of CDK12 activity repressed AR signalling and induced apoptosis in vitro. Collectively, these studies strengthen the view of CRPC as a transcriptionally addicted cancer type and highlight tCDKs as promising targets to prevent oncogenic AR signalling in relapsed tumours.

### TCDK inhibitors as cancer therapeutics

Early drug discovery efforts focused on designing inhibitors that targeted the ATP binding pocket, conserved across the CDK family [62]. The first pan-CDK inhibitor to enter clinical trials was flavopiridol, a semi-synthetic flavone derivative, followed by seliciclib, a purine-based compound [63]. However, the promiscuity of these two inhibitors likely contributed to their failure in the clinic, following numerous patient toxicities and challenges in establishing an accurate pharmacodynamic profile in vivo [63]. The initial setbacks associated with pan-CDK inhibitors paved the foundation for the development of second-generation selective CDK inhibitors. Thus far, CDK4/6 inhibitors (CDK4/6i) have proved the most promising, with three inhibitors (palbociclib, ribociclib, and abemaciclib) currently approved by the U.S. Food and Drug Administration (FDA) for oestrogen receptor (ER)-positive breast cancer treatment. Although beyond the scope of this review, the promising antioncogenic activity combined with the clinical success of CDK4/6i galvanized support for the development of highly selective inhibitors for other CDKs. The next sections will provide an overview of the current tCDK-specific inhibitors that have good drug-like properties and are, therefore, promising candidates for clinical development (Table 2).

**Table 2:** Current status of transcription associated CDK inhibitors in clinical trials.

Name(s)	Inhibitor Type	Intended Therapeutic Target	Major Biologial Target(s) IC_50_ (nM)*	Company	Malignancy Type(s)	Clinical Trial Status
CT7001 (ICEC0942, Samuraciclib)	Non-covalent	CDK7	CDK1 = 1800; CDK2 = 620; CDK4 = 49000; CDK5 = 9400; CDK6 = 34000; **CDK7** = **40**; CDK9 = 1200	Carrick Therapeutics	Advanced Solid Malignancies	Phase I/II NCT03363893 (Active)
				Hoffmann-La Roche	Locally Advanced or Metastatic ER-positive Breast Cancer	Phase I/II NCT04802759 (Recruiting)
SY-1365	Covalent	CDK7	**CDK7** = **20**	Syros Pharmaceuticals	Advanced Solid Tumours	Phase I NCT03134638 (Terminated)
SY-5609	Non-covalent	CDK7	CDK2 = 2900^†^;**CDK7** = **0.06**^**#**^; CDK9 = 970^†^; CDK12 = 770^†^	Syros Pharmaceuticals	Advanced Solid Tumours	Phase I NCT04247126 (Recruiting)
XL102 (AUR102)	Covalent	CDK7	Not disclosed	Exelixis	Advanced Solid Tumours	Phase I NCT04726332 (Recruiting)
LY3405105	–	CDK7	CDK1 = 20000; CDK2 = 20000; CDK4 = 2830; CDK6 = 8079; **CDK7** = **92.8**; CDK9 = 6200; CDK12 = 14780	Eli Lilly and Company	Advanced Solid Tumours	Phase I NCT03770494 (Terminated)
BAY1143572	Non-covalent	CDK9	**CDK9** = **13**; CDK3 = 890; CDK2 = 1000; CDK1 = 1100; CDK5 = 1600	Bayer	Advanced Acute Leukaemia	Phase I NCT02345382 (Completed)
					Advanced Malignancies	Phase I NCT01938638 (Completed)
VIP152 (BAY1251152)	Non-covalent	CDK9	CDK2 = 360; **CDK9** = **3**	Vincerx Pharma	Advanced Hematological Malignancies	Phase I NCT02745743 (Completed)
					Advanced Solid Tumours and Aggressive Non-Hodgkin’s Lymphoma	Phase I NCT02635672 (Recruiting)
					Relapsed/Refractory Chronic Lymphocytic Leukemia or Richter Syndrome	Phase I NCT04978779 (Recruiting)
AZD4573	Non-covalent	CDK9	**CDK9** = < **4**	AstraZeneca	Relapsed/Refractory Haematological Malignances	Phase I NCT03263637 (Completed)
						Phase I/II NCT04630756 (Recruiting)
					Relapsed/Refractory Peripheral T-cell Lymphoma or Classical Hodgkin Lymphoma	Phase II NCT05140382 (Not Yet Recruiting)
KB-0742	Non-covalent	CDK9	**CDK9** = **6**	Kronos Bio	Relapsed or Refractory Solid Tumors; Non-Hodgkin Lymphoma	Phase I NCT04718675 (Recruiting)
GFH009	Not disclosed	CDK9	Not disclosed	GenFleet Therapeutics	Relapsed/Refractory hematologic malignancies	Phase 1 NCT04588922 (Recruiting)
BCD-115 (Senexin B)	Non-covalent	CDK8/19	**CDK8** = **17.3; CDK19** = **4.2**	Biocad	Locally Advanced or Metastatic ER-positive HER2-negative Breast Cancer	Phase 1 NCT03065010 (Completed)
RVU120 (SEL120)	Covalent	CDK8/19	**CDK8** = **4.4; CDK19** = **10.4**	Ryvu Therapeutics	Acute Myeloid Leukemia;High-risk Myelodysplastic Syndrome	Phase I NCT04021368 (Recruting)
					Metastatic or Advanced Solid Tumours	Phase I/II NCT05052255 (Recruting)

*IC_50_ data has been listed for CDK1, 2, 3, 4, 6, 7, 9, and 12 derived from in vitro kinase activity, where available. Data has been omitted from the table if undisclosed or not applicable. ^†^*K*_*d*_ determined by surface plasmon resonance. ^#^*K*_*i*_ determined by activity assay.

*CDK* cyclin-dependent kinase, *ER* Oestrogen receptor.

### CDK7-specific inhibitors

The first highly-selective CDK7 inhibitor, BS-181 was derived from seliciclib using a modelling-based structural design [64]. Preclinical studies using MCF-7 breast cancer cells demonstrated good target engagement, resulting in cell cycle arrest and apoptosis in vitro, and suppression of subcutaneous xenograft growth in vivo [64]. Despite promising antioncogenic effects, poor cell permeability and bioavailability impeded the progression of BS-181 into clinical trials. Nearly 10 years later, structural refinements of BS-181 yielded the first orally bioavailable CDK7 inhibitor, ICEC0942, with 15-230-fold greater selectivity for CDK7 over other CDKs [65]. Preclinical studies showed that ICEC0942 inhibits proliferation in numerous cancer cell types in vitro but also demonstrated that ER-positive cells were especially susceptible to ICEC0942 as a single agent or in combination with endocrine therapies, providing a rationale for the use of CDK7 inhibitors in the treatment of ER-positive breast cancer [65]. ICEC0942/CT7001, now renamed samuraciclib, was licenced to Carrick Therapeutics and is currently in phase I/II clinical studies for advanced solid malignancies as single agent or in combination with standard therapy for specific participant groups, including triple-negative breast cancer (TNBC) and CRPC cohorts (NCT03363893).

THZ1 is a covalent CDK7 inhibitor identified through cell-based screening and kinase selectivity profiling [66]. THZ1 irreversibly targets a cysteine residue (Cys312) located outside of the kinase domain of CDK7, resulting in allosteric inhibition of CDK7 activity. However, THZ1 has a relatively short half-life in vivo [66], and later studies indicated that it covalently inhibits CDK12 and CDK13 in addition to CDK7 [67]. With the aim of improving specificity and stability, several analogues of THZ1 have been developed. A hybrid strategy combining the covalent warhead of THZ1 with a pyrrolidino-pyrazole core produced YKL-5-124. In vitro studies indicated that YKL-5-124 had potent effects on the cell cycle, but did not significantly affect global basal transcription [68]. However, in preclinical models of small cell lung cancer, YKL-5-124 induced genomic instability and triggered an inflammatory response, resulting in potentiation of anti-PD-1 therapy [69]. Although not yet in clinical trials, YKL-5-124 provides a link between CDK7 inhibition and augmented antitumour immunity, and therefore represents a promising new approach in cancer immunotherapy.

Recently, Syros Pharmaceuticals have announced the clinical development of a new orally available ATP-competitive CDK7 inhibitor, SY-5609 [70], after terminating previous studies using the covalent compound SY-1365 [71]. Preclinically, SY-5609 presents with favourable antitumor activity in ER-positive breast cancer [72], TNBC and ovarian cancer models [70]. It is currently being assessed in a phase I dose-escalation study (NCT04247126) in patients with advanced solid tumours, with the most recent update from the company reporting good clinical activity.

AUR102 is a recently described orally bioavailable CDK7-selective inhibitor with preclinical activity in models of breast cancer, prostate cancer, and lymphoma [73]. AUR102, now renamed XL102, was in-licensed by Exelixis from Aurigene and is undergoing clinical testing for advanced or metastatic solid cancers as single-agent or combination therapy (NCT04726332). Another CDK7 inhibitor, LY3405105, developed by Eli Lilly, entered clinical development for patients with advanced solid tumours, however the phase I study was very recently terminated due to lack of efficacy (NCT03770494).

### CDK9-specific inhibitors

Among the first CDK9 inhibitors available, flavopiridol has been studied most extensively. Preclinical evaluations showed potent antiproliferative effects in a range of haematological malignancies [74]. In recent years, more selective CDK9 inhibitors have been developed, of which five were entered into clinical trials: BAY1143572, BAY1251152, AZD4573, KB-0742, and GFH009.

BAY1143572 (atuveciclib), the first highly selective CDK9 inhibitor described by Bayer, exhibited marked tumour growth inhibition in preclinical models of acute myeloid leukaemia [75] and adult T-cell leukaemia/lymphoma [76]. BAY1143572 was trialled in two cohorts of patients with advanced acute leukaemia (NCT02345382) and advanced solid tumours (NCT01938638). However, both studies were terminated early as a therapeutic window could not be identified, and the clinical development program for BAY1143572 was discontinued. Bayer also sponsored two phase I studies using BAY1251152 (later renamed VIP152), a follow-up more potent CDK9 inhibitor. The first trial, in patients with advanced blood cancer, failed to show clinical efficacy despite evidence of target engagement (NCT02745743) [77]. The second trial (NCT02635672) showed a manageable safety profile and signs of antitumour activity [78], particularly in MYC-driven lymphoma and solid tumours [79]. This study is still ongoing, although sponsorship was transferred to Vincerx Pharma. Additionally, Vincerx Pharma recently announced a new phase I trial to evaluate VIP152 in relapsed/refractory chronic lymphocytic leukaemia or Richter’s Transformation (NCT04978779).

A structure-based approach led to the identification of AZD4573, a highly potent CDK9 inhibitor with over 25-fold selectivity for CDK9 over other CDKs, and broad antitumor activity across preclinical hematologic cancer models [80, 81]. A phase I trial of AZD4573 in patients with relapsed or refractory haematological malignancies was recently completed (NCT03263637, pending results reporting), while a phase I/II study has recently started recruiting patients with advanced blood cancers to be treated with AZD4573 in combination with acalabrutinib (NCT04630756). In addition, a phase II trial exploring the safety and efficacy of AZD4573 as monotherapy or in combination with anti-cancer agents for patients with relapsed/refractory peripheral T-cell lymphoma or classical Hodgkin lymphoma was announced at the end of 2021 (NCT05140382).

The CDK9 inhibitors mentioned have short in vivo half-lives (<1 h) and limited activity in preclinical models of solid tumours, therefore they are primarily trialled being against haematological cancers. Kronos Bio’s recently discovered KB-0742, a well-tolerated, orally bioavailable CDK9 inhibitor, significantly reduces tumour burden in models of prostate cancer in vivo [59]. This provided compelling support for developing KB-0742 as therapeutic for solid cancers. Encouragingly, interim analysis of Kronos Bio’s ongoing phase I/II trial of KB-0742 (NCT04718675) demonstrated dose-dependent target engagement and a terminal half-life of 24 h in patients with advanced solid tumours.

Limited information is available about GenFleet Therapeutics’ highly selective CDK9 inhibitor, GFH009, other than the announcement of a phase I clinical trial for haematological malignancies (NCT04588922).

### CDK12/13-specific inhibitors

Three CDK12/13-specific inhibitors have been described, although none have progressed into clinical evaluation. THZ531 and the optimised BSJ-01-175, both derived from THZ1, are covalent CDK12/13 inhibitors [67, 82], while SR-4835 is an reversible ATP competitive inhibitor [83]. To date, in vivo preclinical studies using these inhibitors showed antitumour activity in models of Ewing sarcoma and TNBC, and demonstrated that CDK12/13 inhibition may improve the efficacy of DNA-damaging chemotherapy and PARP inhibitors [82, 83]. Notably, deleterious loss-of-function *CDK12* mutations are frequently observed in cancer, including in 3-7% of patients with metastatic CRPC [84]. While associated with aggressive disease and poor prognosis, *CDK12* alterations also induce a BRCAness phenotype due to perturbation of in the homologous recombination repair pathway. However, in a recent study, the transcriptional alterations mediated by small molecule CDK12/13 inhibitors differed from those mediated by loss-of-function *CDK12* mutations [61], suggesting selective CDK12/13 inhibitors may function through a distinct mechanism, which warrants further exploration.

### CDK8/19-specific inhibitors

Cortistatin A is a naturally occurring compound identified as the first selective CDK8 inhibitor in 2015 [37]. Since then, numerous small molecule CDK8/19 inhibitors have been described, including Cmpd3 and Cmpd4, Senexin B, and SEL120. Preclinical studies using Cmpd3 and Cmpd4, two chemically distinct CDK8/19 inhibitors, showed encouraging antitumour activity in a number of tumour models but poor toxicity profile, with considerable weight loss and multiple systemic toxicities, including lethality [85]. This raised concerns that inhibiting CDK8/19 does not provide sufficient therapeutic window, although later reports suggested the adverse events to be unrelated to CDK8/19 inhibition, pointing to off-target inhibition as the most likely cause of toxicity [86]. In 2017, Senexin B (renamed BCD-115) was the first selective CDK8/19 inhibitor to enter a phase I clinical trial which was completed (NCT03065010). The compound was evaluated for the treatment of women with ER-positive, human epidermal growth factor receptor-2 (HER2)-negative, locally advanced or metastatic breast cancer.

SEL120 is an orally bioavailable, highly potent, ATP-competitive CDK8/19 inhibitor which supresses growth in vivo in models of acute myeloid leukaemia [87]. In April 2020, the FDA granted orphan drug status to SEL120 (renamed RVU120) for the treatment of patients with acute myeloid leukaemia. The first trial using RVU120 began in 2019 (NCT04021368) but was paused April 2021 due to reports of a serious adverse effect potentially related to RVU120 administration that resulted in the death of a patient. In July 2021, the FDA lifted the partial clinical hold on this study and Ryvu Therapeutics, the sponsoring company, announced the beginning of parallel phase I/II study of RVU120 as single agent in patients with relapsed or refractory metastatic or advanced solid tumours (NCT05052255).

## Emerging combination strategies using tCDK inhibitors

**Fig. 3:**
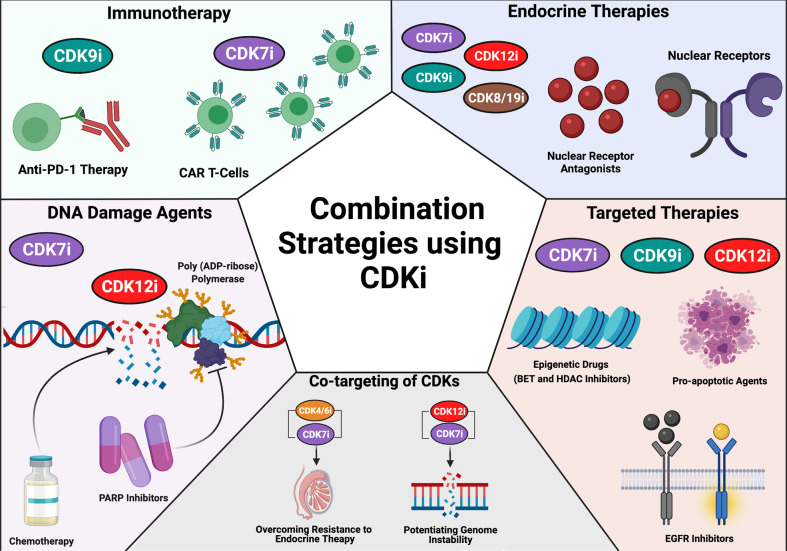
Potential combination strategies with transcription associated CDK inhibitors (CDKi) for the treatment of advanced malignancies. PD-1, programmed cell death protein 1; CAR, chimeric antigen receptor; PARP, poly (ADP-ribose) polymerase; BET, bromodomain and extra-terminal motif; HDAC, histone deactylase; EGFR, epidermal growth factor receptor. Created with BioRender.com.

Although many of the inhibitors mentioned have shown significant antitumour activity as single agents, the dynamic nature of advanced tumours often demands inhibition of multiple targets to limit disease reoccurrence. As such, efforts have also focused on developing tCDK inhibitors as adjuvant therapies (Fig. 3). Finding efficacious combination strategies with established anticancer therapies can provide additive or synergistic activity, allowing dose reduction and minimising the risk of treatment-related toxicity. To date, none of the strategies below have been trialled in prostate cancer, but some are already undergoing clinical assessment in other cancer types.

### Endocrine therapies

Due to the role of tCDKs in modulating the transcriptional activity of hormone receptors, a potentially promising therapeutic strategy is combining tCDK inhibitors with endocrine therapies. Preclinical studies of CT7001 in breast cancer models provided evidence that the combination of CDK7 inhibitors with tamoxifen is superior to either monotherapy [65]. In August 2021, Carrick Therapeutics announced it was granted Fast Track designation for CT7001 in combination with the antioestrogen fulvestrant for the treatment of CDK4/6i-resistant hormone receptor-positive, HER2-negative advanced breast cancer and that it will begin evaluating CT7001 in combination with giredestrant, a next-generation selective oestrogen receptor degrader, in collaboration with Roche (NCT04802759).

Preclinical evidence also supports combination use of tCDK inhibitors with antiandrogens in prostate cancer. Inhibition of CDK12 was shown to synergise with antiandrogens through a mechanism involving decreased acetylation of histone H3K27 at AR:FOXA1 binding sites [61]. In addition, CDK7, CDK9 and CDK8/19 inhibitors are potential candidates for use in combination with antiandrogens, since inhibition of these kinases was shown to suppress AR signalling in disease-relevant models [55, 59, 60].

### DNA and DNA-repair targeted therapies

The activity of CDK7 and CDK12/13 are fundamental to maintaining genome stability and repairing DNA damage following genomic insult. Consistently, CDK7 inhibition by YKL-5-124 has been shown to elicit genomic instability in small cell lung cancer models, while CDK12/13 inhibition has been shown to provoke a “BRCA-ness” phenotype in TNBC [69, 83]. Furthermore, CDK7 inhibition synergises with p53-activating agents, including 5-fluorouracil, and induces apoptosis in colorectal cancer cells [88], while combining CDK12/13 inhibition with DNA-damaging chemotherapy, including cisplatin, irinotecan, doxorubicin and with the PARP inhibitor olaparib was shown to augment anticancer activity in TNBC cells [83]. Consequently, it is foreseeable that combining CDK7 and CDK12/13 inhibitors alongside DNA damage-inducing agents may further promote genomic instability. Carrick Therapeutics announced it was granted Fast Track designation for the CDK7 inhibitor CT7001 in combination with chemotherapy for the treatment of locally advanced or metastatic TNBC, while Syros Pharmaceuticals announced it will begin clinical investigation of the CDK7 inhibitor SY-5609 in combination with chemotherapy in pancreatic cancer [89].

With respect to potential similar combinations in prostate cancer, docetaxel is the primary chemotherapy administered to patients with metastatic CRPC. Very recently, the FDA also approved the use of two PARP inhibitors for the treatment of patients with metastatic CRPC who harbour DNA damage response gene mutations (particularly *BRCA* deficiency). However, to date, there are no published preclinical data supporting combinatorial use of tCDK inhibitors with DNA damaging or DNA repair-targeted agents in prostate cancer models.

### Immunotherapies

There has also been a growing interest in the use of tCDK inhibitors in potentiating immunotherapies. A number of studies have now indicated CDK7 and CDK9 play a role in maintaining genome stability, and inhibition of their activity may sensitise tumours to immune checkpoint inhibitors. Therefore, the use of CDK7 and CDK9 inhibitors in combination with immune checkpoint inhibitors may represent a powerful therapeutic combination, particularly for tumours displaying a “cold” immune microenvironment, as is the case of prostate cancer. In line with this, the genomic instability induced by CDK7 inhibition in small cell lung cancer models activated antitumour T-cells, eliciting a robust immune surveillance program which improved the efficacy of anti-PD-1 therapy [69]. Similarly, CDK9 inhibition sensitised a syngeneic ovarian cancer model to anti-PD-1 therapy, by derepressing endogenous retroviruses and inducing an interferon response [90]. Syros Pharmaceuticals recently announced it will begin investigating the CDK7 inhibitor SY-5609 in combination with atezolizumab in patients with BRAF-mutant colorectal cancer [89].

Evidence also suggests tCDK inhibitors may be useful as neoadjuvant therapy preceding chimeric antigen receptor (CAR) engineered T-cells therapy. Cytokine release syndrome is the most common toxicity observed following CAR T-cell therapy. Pre-treatment of tumour-bearing mice with THZ1, a CDK7/CDK12/13 inhibitor, before infusion with CAR T-cells attenuated cytokine release by suppressing transcription of inflammatory genes, thus preventing the associated systemic toxicity [91].

### Other targeted therapies

Receptor tyrosine kinase inhibitors used in combination with tCDK inhibitors include epidermal growth factor receptor (EGFR) and HER2 inhibitors. In breast cancer models, the EGFR inhibitor erlotinib displayed synergy with THZ1 and with the dual cdc7/CDK9 inhibitor PHA-767491 [92, 93], with more lines of evidence suggesting CDK7 and CDK9 inhibitors could sensitise resistant cancer cells to EGFR-targeted therapy [93, 94]. A similar effect was seen with THZ1 and the HER2 inhibitor lapatinib, which displayed synergistic effects when combined in breast cancer models independently of HER2 inhibitor-resistance status [95].

Venetoclax is a selective inhibitor of the anti-apoptotic protein B-cell lymphoma 2 (Bcl-2) currently used in haematological cancers displaying increased Bcl-2 expression. Venetoclax has been investigated in combination with several CDK9-specific inhibitors, including AZD4573 and A-1592668, and voruciclib, a flavopiridol-derivative with improved specificity for CDK9, with combination therapy showing superior efficacy to either agent alone [80, 96, 97]. Mechanistically, it has been suggested that CDK9 inhibitors enhance venetoclax activity by inhibiting transcription of anti-apoptotic proteins, thereby inducing apoptosis more efficiently [80]. In addition to CDK9 inhibitors, the selective CDK7 inhibitor SY-1365 also synergised with venetoclax in haematological cancers through a similar mechanism involving downregulation of anti-apoptotic proteins [71].

Recent work has also highlighted the potential use of tCDK inhibitors in combination with epigenetic drugs, including Bromodomain and Extra-Terminal (BET) motif protein inhibitors (e.g., JQ1, which synergises with CDK7 inhibition in models of ovarian cancer [98], neuroblastoma [99], and iBET-151, which synergises with CDK9 inhibition in models of leukaemia [100]), and histone deacetylase inhibitors (e.g., panobinostat, which synergised with THZ1 in models of diffuse intrinsic pontine glioma [101]). In addition, combination of BRD4 inhibitors with CDK7 or CDK9 inhibitors is envisaged to effectively target AR super-enhancer addiction in CRPC [102], although preclinical studies addressing this are currently lacking.

### Co-targeting of CDKs

Although limited evidence is available to date, exploring the ability of CDK inhibitors to act complementarily to one another is highly warranted. This is especially promising where different CDKs function together to modulate fundamental cellular processes, as is the case of the Pol II CTD kinases CDK7, CDK9 and CDK12/13 regulating transcription. In line with this, co-targeting of CDK7 and CDK9 synergistically downregulated expression of the oncogene MYC and the anti-apoptotic protein MCL-1 and induced apoptosis through p53 activation in models of acute myeloid leukaemia [103]. In metastatic prostate cancer displaying transcriptional addiction, it can be envisaged that treatment with a combination of CDK7, CDK9, and/or CDK12/CDK13 inhibitors may lead to potent suppression of oncogenic transcription, since all of these tCDKs have established AR signalling and tumour-promoting roles in relevant disease models [55, 59, 61].

In addition, co-targeting of CDK7 along with cell cycle CDKs may be a strategy to prevent therapy resistance. A genome-wide CRISPR knock-out screen in parental and CDK4/6i-resistant hormone receptor-positive breast cancer cell lines identified *CDK4*, *CDK6*, and *CCND1* as essential genes in the parental line but not in the CDK4/6i-resistant line, in which one of the top-ranked essential genes was CDK7 [104].

## Conclusions

In summary, tCDKs have central roles in efficient gene transcription. As well as being a requisite process in all cells, transcription is often hyperactive in many cancer types, which could be described as transcriptionally addicted. Therefore, tCDKs are emerging as promising cancer therapeutic targets and selective tCDK inhibitors have rapidly advanced in clinical trials as single agents against a wide range of tumour types. Although these inhibitors are rapidly becoming accepted as safe and specific ways to target cancer cells, clinical trial results reported to date have been mixed. This is in part due to the suboptimal selectivity profiles of first-generation tCDK inhibitors, but may also be due to incomplete understanding of the genetic and microenvironmental factors that influence tumour response to tCDK inhibition. Moreover, possible resistance mechanisms that may emerge as result of selective targeting of tCDKs are only now beginning to be identified. Notwithstanding, the potential for more selective compounds, coupled with the demonstrations that tCDK inhibitors display promising complementary activity to existing cancer therapeutics, has substantially boosted interest in the field, with several synergistic drug combinations showing potential to translate into clinical use in the near future.
